# Type 2 Diabetes–Prevention Diet and All-Cause and Cause-Specific Mortality: A Prospective Study

**DOI:** 10.1093/aje/kwab265

**Published:** 2021-11-02

**Authors:** Chun-Rui Wang, Tian-Yang Hu, Fa-Bao Hao, Nan Chen, Yang Peng, Jing-Jing Wu, Peng-Fei Yang, Guo-Chao Zhong

**Keywords:** mortality, primary prevention, prospective study, type 2 diabetes–prevention diet

## Abstract

We aimed to examine whether type 2 diabetes–prevention diet, a dietary pattern previously developed for reducing type 2 diabetes risk, was associated with mortality in a US population. A population-based cohort of 86,633 subjects was identified from the Prostate, Lung, Colorectal, and Ovarian Cancer Screening Trial (1993–2015). Dietary information was collected with a food frequency questionnaire. A dietary diabetes risk-reduction score was calculated to reflect adherence to this dietary pattern, with higher scores representing better adherence. Hazard ratios (HRs) and absolute risk differences (ARDs) in mortality rates per 10,000 person-years were calculated. After a mean follow-up of 13.6 years, 17,532 all-cause deaths were observed. Participants with the highest versus the lowest quintiles of dietary diabetes risk-reduction score were observed to have decreased risks of death from all causes (HR = 0.76, 95% CI: 0.72, 0.80; ARD: −81.94, 95% CI: −93.76, −71.12), cardiovascular disease (HR = 0.73, 95% CI: 0.66, 0.81; ARD: −17.82, 95% CI: −24.81, −11.30), and cancer (HR = 0.85, 95% CI: 0.78, 0.94; ARD: −9.92, 95% CI: −15.86, −3.59), which were modified by sex, smoking status, or alcohol consumption in subgroup analyses (*P* for interaction < 0.05 for all). In conclusion, a type 2 diabetes–prevention diet confers reduced risks of death from all causes, cardiovascular disease, and cancer in this US population.

## Abbreviation

CI: confidence interval
DHQ: diet history questionnaire
HR: hazard ratio
PLCO: Prostate, Lung, Colorectal, and Ovarian

Type 2 diabetes is a major public health concern worldwide and is a well-established predisposing factor for cardiovascular disease (1) and cancer (2), which represent 2 leading global causes of death. Dietary behaviors play a critical role in public health; unhealthy diet is ranked as the most common cause of death in the US population (3). Hence, it is essential to investigate the potential associations of dietary behaviors with health outcomes.

A type 2 diabetes–prevention diet was proposed by Rhee et al. in 2015 (4) and features high intakes of cereal fiber, polyunsaturated fatty acids, coffee, and nuts and low intakes of carbohydrates, *trans*-fatty acids, red and processed meat, and sugar-sweetened beverages (4). Compared with other established dietary patterns (e.g., the Mediterranean diet), the type 2 diabetes–prevention diet captures key dietary elements closely related to the risk of type 2 diabetes and is developed primarily for facilitating the prevention of this disease (4), resulting in inclusion of some components that are not part of other established dietary patterns (e.g., coffee and glycemic index); moreover, adherence to the type 2 diabetes–prevention diet could improve insulin sensitivity and reduce inflammation levels (4–6). Recently, the type 2 diabetes–prevention diet was found to be associated with reduced risks of hepatocellular carcinoma (7), breast cancer (8), and pancreatic cancer (9). However, whether the type 2 diabetes–prevention diet is associated with mortality remains unknown. Some studies have investigated the associations between individual components of the type 2 diabetes–prevention diet with the risk of mortality (10–16), but they fail to consider the potential interactions among dietary components. Therefore, assessment of dietary patterns, which include multiple foods or nutrients simultaneously and thus can capture the potential interactions among them, may provide a more accurate estimate for diet-disease associations.

Hence, in this study, we aimed to examine the hypothesis that adherence to the type 2 diabetes–prevention diet is associated with all-cause and cause-specific mortality in the US population.

## METHODS

The results of the present study were reported in accordance with the Strengthening the Reporting of Observational Studies in Epidemiology statement (17).

### Study population

Our study population was identified from the Prostate, Lung, Colorectal, and Ovarian (PLCO) Cancer Screening Trial, a large randomized clinical study with 10 enrollment centers (St. Louis, Missouri; Honolulu, Hawaii; Denver, Colorado; Pittsburgh, Pennsylvania: Marshfield, Wisconsin; Birmingham, Alabama; Salt Lake City, Utah; Washington, DC; Minneapolis, Minnesota; and Detroit, Michigan). This trial was designed to investigate the potential beneficial effects of selected screening exams on the risks of death from prostate, lung, colorectal, and ovarian cancers. Study design of the PLCO Cancer Screening Trial has been reported elsewhere (18). Briefly, during November 1993 and September 2001, individuals aged 55–74 years were invited to take part in this trial. A total of 154,887 individuals were qualified for enrollment and individually randomized to the intervention group or the control group in equal proportions, with individuals in the intervention group receiving selected screening exams while those in the control group received usual care. All participants provided written informed consent. The PLCO Cancer Screening Trial was approved by the institutional review boards of the US National Cancer Institute and each enrollment center.

**Figure 1:**
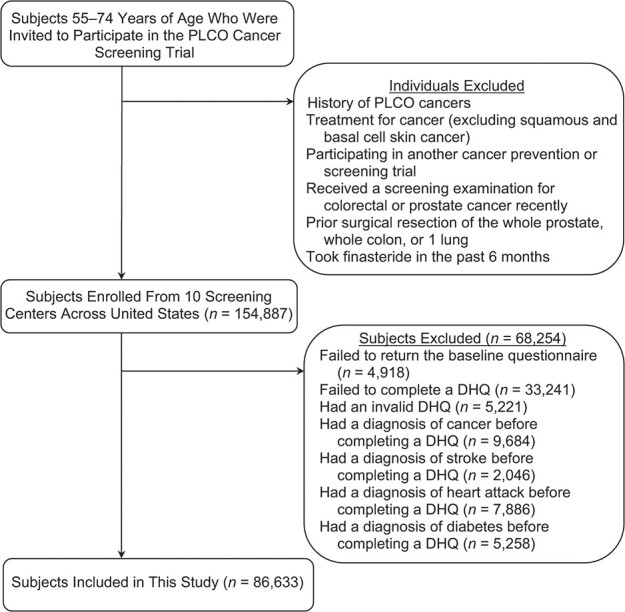
Flow chart identifying subjects included in this study evaluating a type 2 diabetes–prevention diet and multiple causes of mortality, a post hoc analysis of the Prostate, Lung, Colorectal, and Ovarian (PLCO) Cancer Screening Trial, United States, 1993–2009. The total number of subjects for an exclusion category box was not available in the PLCO Cancer Screening Trial. DHQ, diet history questionnaire.

The following participants were further excluded from our study: 1) 4,918 participants failing to return a baseline questionnaire, a baseline risk-factor questionnaire with participant-reported information (e.g., demographic characteristics and medical history); 2) 33,241 participants failing to return a diet history questionnaire (DHQ); 3) 5,221 participants with an invalid DHQ—the valid DHQ refers to having a DHQ completion date, DHQ completion date prior to death date, <8 missing frequency responses, and the absence of extreme energy intake (top 1% and bottom 1%); 4) 9,684 participants with a history of cancer at baseline; 5) 2,046 participants with a history of stroke at baseline; 6) 7,886 participants with a history of heart attack at baseline; and 7) 5,258 participants with a history of diabetes at baseline. Finally, a total of 86,633 participants were included (Figure 1). The reason for excluding participants with a history of cancer, stroke, heart attack, or diabetes at baseline was that they might alter their dietary habits after receiving these diagnoses, which might result in reverse causation.

### Calculation of dietary diabetes risk-reduction score

A dietary diabetes risk-reduction score was calculated to quantify adherence to a type 2 diabetes–prevention diet using the approach described in the literature (4). Briefly, all participants were divided into 5 strata based on quintiles of dietary intake of each component. For favorable components (i.e., cereal fiber, ratio of polyunsaturated to saturated fatty acids, coffee, and nuts), participants in the highest stratum were awarded 5 points and those in the lowest stratum were awarded 1 point; in contrast, for unfavorable components (i.e., glycemic index, *trans*-fatty acids, red and processed meat, and sugar-sweetened beverages), participants in the highest stratum were awarded 1 point and those in the lowest stratum were awarded 5 points (Web Table 1, available at https://doi.org/10.1093/aje/kwab265). An individual’s dietary diabetes risk-reduction score was calculated as the sum of points for each dietary component, with a range of 8–40 points. Higher scores suggest greater adherence to the diet. Glycemic index was calculated as described previously (19). Notably, in this study, sugar-sweetened beverages referred to soft drinks or fruit drinks, and cereal fiber referred to insoluble fiber. In addition, given that higher consumption of fruits and vegetables has been identified to be associated with a lower risk of type 2 diabetes (20), we calculated a modified dietary diabetes risk-reduction score by regarding these 2 foods as favorable components (Web Table 2).

In the PLCO Cancer Screening Trial, food or nutrient intakes, including those used for the calculation of dietary diabetes risk-reduction score, were evaluated at the study baseline through the DHQ. The DHQ is a 137-item self-administered food frequency questionnaire designed for evaluating food and supplement consumption over the past year; its validity had been confirmed elsewhere (21). Daily food consumption for each participant was estimated by multiplying food frequency by serving size; daily nutrient intake was calculated based on 2 nutrient databases, namely US Department of Agriculture’s 1994–1996 Continuing Survey of Food Intakes by Individuals (22) and Nutrition Data Systems for Research (23).

### Outcome assessment

Mortality status of each participant was confirmed predominantly through a mailed annual study update form. Participants failing to return this form were contacted repeatedly by telephone or e-mail. Moreover, mortality status was adjudicated by periodic linkage to the US National Death Index. The ninth revision of *International Classification of Diseases* was applied to define the underlying causes of death obtained from death certificates: cardiovascular disease (codes 390–459) and cancer (codes 140–209).

### Covariate assessment

Age at DHQ completion, alcohol consumption, single or multivitamin supplement use, and food consumption were collected with the above-mentioned DHQ. Of note, dietary intakes of foods and nutrients were adjusted for energy intake from diet with the residual approach (24) before data analysis. Physical activity level was defined as total time of moderate to vigorous activity per week, and was assessed through a self-administered supplemental questionnaire. Healthy Eating Index 2015 and the plant-based diet index were computed as described in the literature (25, 26). Sex, ethnic group, marital status, body weight, height, educational level, smoking status, history of hypertension, family history of cancer, and aspirin use were collected with a self-administered baseline questionnaire. Body mass index was calculated as body weight (kg) divided by height squared (m^2^).

### Statistical analysis

To minimize potential biases and maximize statistical power, multiple imputation with chained equations was applied to impute missing data under the assumption that data were missing at random (the number of imputations = 25) (27); all variables involved in data analysis were applied to yield imputed data sets. Web Table 3 shows the distribution of covariates with missing values before and after multiple imputation. Main data analyses were repeated for participants with complete data to determine the potential influences of data imputation on our results.

**Figure 2:**
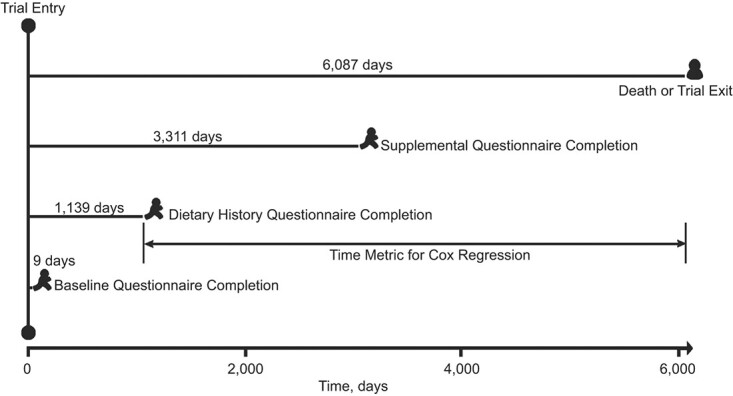
The timeline and follow-up scheme for this study evaluating a type 2 diabetes–prevention diet and multiple causes of mortality, a post hoc analysis of the Prostate, Lung, Colorectal, and Ovarian Cancer Screening Trial, United States, 1993–2009. Note that the time span between 2 events represents the average value of all subjects.

To evaluate the associations of the dietary diabetes risk-reduction score with all-cause and cause-specific mortality, hazard ratios (HRs) and 95% confidence intervals (CIs) were calculated using a Cox proportional hazards regression model, with follow-up time as time metric. In our study, follow-up time was calculated as the difference between DHQ completion date and death date, loss to follow-up, or the end of follow-up (December 31, 2015), whichever came first (Figure 2). In regression models, the dietary diabetes risk-reduction score was split into quintiles, with the first quintile as the reference group. For examining linear trends in risk estimates across quintiles of dietary diabetes risk-reduction score, the median of each quintile was assigned to each participant in the quintile at first to yield an ordinal variable, which was then treated as a continuous variable in regression models for testing its significance. No evidence suggesting the violation of the proportional hazards assumption was found, using the Schoenfeld residuals method (all *P* values for global test >0.05). Covariate selection for multivariable regression was based on the change-in-estimate approach (28) and our knowledge of the existing literature. Specifically, model 1 adjusted for age and sex; model 2 further adjusted for ethnic group, trial arm, educational level, marital status, history of hypertension, family history of cancer (only for all-cause and cancer mortality), aspirin use, single or multivitamin supplement use, smoking status, alcohol consumption, body mass index, physical activity, and energy intake from diet; and model 3 further adjusted for consumption of fruits, vegetables, tea, fish, and dairy. We also performed an analysis treating body mass index as a time-varying covariate (model 4). Moreover, we also calculated absolute risk difference in mortality rate per 10,000 person-years for each HR from the above Cox regression analysis and the below subgroup analysis using the method described in the literature (29).

Prespecified subgroup analyses were performed after stratifying for age (≥65 vs. <65 years), sex (male vs. female), trial group (intervention group vs. control group), history of hypertension (yes vs. no), body mass index (≥25 vs. <25), smoking status (current or past vs. never), and alcohol consumption (heavy vs. no, light, or moderate). For men, we defined light, moderate, and heavy alcohol consumption as ≤6 g/day, >6 and ≤28 g/day, and >28 g/day, respectively; for women, we defined light, moderate, and heavy alcohol consumption as ≤6 g/day, >6 and ≤14 g/day, and >14 g/day, respectively (30). A *P* for interaction was estimated by comparing models with and without multiplicative interaction terms prior to performing the above-mentioned subgroup analyses to avert the possible spurious subgroup differences.

Sensitivity analyses were performed to determine the stability of our results: 1) including participants with a history of cancer, stroke, heart attack, or diabetes at baseline; 2) excluding deaths observed within the first 5 years of follow-up to determine the possibility of the observed association resulted from reverse causation; 3) excluding participants with implausible energy intake from diet, defined as <800 or >4,000 kcal/day for men and <500 or >3,500 kcal/day for women (31); 4) repeating analyses with a competing risk regression model (only for cause-specific mortality) to evaluate the potential influences of competing risk bias; 5) adjustment for propensity score on crude model (all covariates included in model 3 were applied to calculate propensity score with logistic regression); 6) additionally adjusting for Healthy Eating Index 2015 or plant-based index in model 3 to test whether the observed associations were mediated by diet quality, and 7) additionally adjusting for intakes of polyunsaturated and saturated fatty acids per reviewer’s suggestion.

**Table 1:** Baseline Characteristics of Study Population According to Quintiles of Dietary Diabetes Risk-Reduction Score, a Post Hoc Analysis of the Prostate, Lung, Colorectal, and Ovarian Cancer Screening Trial, United States, 1993–2015

	**Quintiles of Dietary Diabetes Risk Reduction Score, Range (Median)**														
	**9–19 (17)** **(*n* = 16,302)**			**20–22 (21)** **(*n* = 17,623)**			**23–24 (23)** **(*n* = 13,339)**			**25–27 (26)** **(*n* = 18,023)**			**28–40 (30)** **(*n* = 21,346)**		
**Characteristic**	**Mean (SD)**	**No.**	**%**	**Mean (SD)**	**No.**	**%**	**Mean (SD)**	**No.**	**%**	**Mean (SD)**	**No.**	**%**	**Mean (SD)**	**No.**	**%**
Age, years	64.5 (5.6)			65.1 (5.7)			65.3 (5.7)			65.5 (5.7)			65.6 (5.7)		
Male sex		9,745	59.8		8,962	50.9		6,176	46.3		7,330	40.7		7,463	35.0
Ethnicity															
Non-Hispanic White		14,982	91.9		16,316	92.6		12,263	91.9		16,470	91.4		19,241	90.1
Non-Hispanic Black		785	4.8		552	3.1		341	2.6		416	2.3		454	2.1
Hispanic		239	1.5		237	1.3		172	1.3		246	1.4		310	1.5
Others^a^		296	1.8		518	2.9		563	4.2		891	4.9		1,341	6.3
Married or living as married		13,082	80.2		14,052	79.7		10,611	79.5		14,191	78.7		16,197	75.9
Body mass index^b^	28.0 (4.9)			27.5 (4.8)			27.1 (4.6)			26.7 (4.5)			25.8 (4.3)		
Educational level															
Some college or below		11,753	72.1		11,762	66.7		8,490	63.6		10,870	60.3		11,604	54.4
College graduate		2,377	14.6		2,969	16.8		2,448	18.4		3,411	18.9		4,362	20.4
Postgraduate		2,172	13.3		2,892	16.4		2,401	18.0		3,742	20.8		5,380	25.2
Alcohol consumption, g/day	7.6 (20.4)			8.7 (21.6)			9.7 (24.3)			10.7 (26.9)			11.6 (30.1)		
Smoking status															
Current		2,114	13.0		1914	10.9		1,248	9.4		1,451	8.1		1,272	6.0
Past		6,316	38.7		7,040	39.9		5,556	41.7		7,573	42.0		9,448	44.3
Never		7,872	48.3		8,669	49.2		6,535	49.0		8,999	49.9		10,626	49.8
Physical activity, minutes/week^c^	107.0 (117.2)			114.1 (120.0)			120.5 (119.7)			126.9 (121.9)			145.8 (129.4)		
Energy intake from diet, kcal/day	1937.8 (767.6)			1790.4 (769.0)			1697.6 (739.5)			1646.8 (711.3)			1639.6 (653.4)		
HEI-2015 score	56.5 (7.9)			62.7 (7.5)			66.3 (7.2)			69.5 (7.0)			75.1 (6.8)		
Plant-based diet index score	52.5 (5.9)			53.4 (6.3)			53.5 (6.4)			53.9 (6.5)			55.0 (6.5)		
History of hypertension		5,161	31.7		5,435	30.8		3,927	29.4		5,110	28.4		5,449	25.5
Family history of cancer		9,082	55.7		9,906	56.2		7,465	56.0		10,310	57.2		12,088	56.6
Aspirin use															
Yes		6,909	42.4		7,682	43.6		5,771	43.3		7,952	44.1		9,328	43.7
No		9,393	57.6		9,941	56.4		7,568	56.7		10,071	55.9		12,018	56.3
Single or multivitamin supplement use															
Yes		11,031	67.7		13,183	74.8		10,414	78.1		14,612	81.1		18,506	86.7
No		5,271	32.3		4,440	25.2		2,925	21.9		3,411	18.9		2,840	13.3
Energy-adjusted food and nutrient intakes															
Glycemic index	56.6 (3.6)			54.8 (3.8)			53.7 (3.8)			52.6 (3.8)			50.7 (3.7)		
Cereal fiber, g/day	9.1 (4.8)			10.4 (5.5)			11.2 (5.9)			12.1 (6.2)			15.1 (7.2)		
Ratio of polyunsaturated to saturated fatty acids	0.6 (0.5)			0.7 (0.2)			0.7 (0.3)			0.8 (0.5)			1.0 (0.4)		
*Trans*-fatty acids, g/day	6.0 (3.4)			4.9 (3.2)			4.1 (2.8)			3.3 (2.5)			2.1 (1.9)		
Sugar-sweetened beverages, g/day	537.9 (705.7)			283.7 (436.3)			194.3 (349.0)			138.5 (263.2)			78.4 (186.6)		
Nuts, g/day	1.9 (4.8)			3.8 (8.3)			5.0 (10.4)			7.1 (13.8)			13.9 (24.6)		
Coffee, g/day	654.1 (769.9)			827.5 (806.9)			875.6 (800.0)			907.6 (788.9)			950.2 (778.4)		
Red and processed meat, g/day	22.0 (20.8)			15.4 (17.3)			11.7 (13.9)			8.9 (11.4)			4.8 (7.9)		
Polyunsaturated fatty acids, g/day	14.8 (7.7)			14.4 (8.3)			13.7 (8.2)			13.5 (8.2)			14.0 (8.4)		
Saturated fatty acids, g/day	26.8 (13.8)			22.7 (13.1)			19.8 (11.6)			17.6 (10.5)			14.5 (8.4)		
Fruit, g/day	199.2 (172.0)			236.1 (185.8)			260.1 (200.9)			285.3 (212.5)			357.6 (251.5)		
Vegetable, g/day	237.8 (146.9)			258.4 (163.0)			267.1 (173.0)			285.2 (179.0)			344.3 (222.3)		
Tea, g/day	277.1 (515.5)			258.2 (469.8)			253.5 (453.2)			254.1 (453.4)			263.4 (441.7)		
Fish, g/day	14.5 (19.1)			14.3 (17.2)			14.7 (18.2)			15.4 (19.1)			17.4 (21.9)		
Dairy, servings/day	1.3 (2.1)			1.4 (2.2)			1.4 (2.1)			1.4 (2.1)			1.4 (2.0)		

Abbreviations: HEI, Healthy Eating Index; SD, standard deviation.

^a^ “Others” refers to Asian, Pacific Islander, or American Indian.

^b^ Weight (kg)/height (m)^2^.

^c^ Total time of moderate to vigorous physical activity per week.

To determine the main contributor(s) of the type 2 diabetes–prevention diet, we examined the association between each component of this dietary pattern and the risk of death separately. Statistical analyses were conducted with STATA software (version 12.0; StataCorp LP, College Station, Texas). The statistical significance level was set at *P* < 0.05 under a 2-tailed test.

## RESULTS

### Participant characteristics

Participants in the highest versus the lowest quintiles of dietary diabetes risk-reduction score were less likely to be male, be married or living as married, be current smokers, and have a history of hypertension but more likely to be single or multivitamin supplement users, have lower body mass index and energy intake from diet and had higher educational level, alcohol consumption, physical activity level, and Healthy Eating Index 2015 (Table 1). In addition, compared with participants in the lowest quintile of dietary diabetes risk-reduction score, those in the highest quintile had lower glycemic index and lower intakes of *trans*-fatty acids, sugar-sweetened beverages, red and processed meat, and saturated fatty acids but higher intakes of cereal fiber, nuts, coffee, fruits, and vegetables.

### Dietary diabetes risk-reduction score and all-cause and cause-specific mortality

During 1,174,401.6 person-years of follow-up, we observed a total of 17,532 all-cause deaths, of which 4,809 (27.4%) were attributable to cardiovascular disease and 5,719 (32.6%) to cancer (Table 2). The mean follow-up was 13.6 (standard deviation, 3.2) years. The crude death rates per 10,000 person-years were 149.28, 40.95, and 48.70 for mortality from all causes, cardiovascular disease, and cancer, respectively, which were obviously lower than those from the National Institutes of Health–AARP study, a contemporary US cohort study involving 521,120 participants (176.99, 53.03, and 62.66 deaths per 10,000 person-years for mortality from all causes, cardiovascular disease, and cancer, respectively) (32). In the fully adjusting model, participants in the highest (5th) vs. the lowest (1st) quintiles of dietary diabetes risk-reduction score were found to be at lower risks of death from all causes (HR = 0.76, 95% CI: 0.72, 0.80; *P* for trend< 0.001; absolute risk difference = −81.94, 95% CI: −93.76, −71.12), cardiovascular disease (HR = 0.73, 95% CI: 0.66, 0.81; *P* for trend < 0.001; absolute risk difference = −17.82, 95% CI: −24.81, −11.30), and cancer (HR = 0.85, 95% CI: 0.78, 0.94; *P* for trend < 0.001; absolute risk difference = −9.92, 95% CI: −15.86, −3.59) (Table 2 and Web Table 4). We obtained similar results when repeating the above-mentioned Cox regression analyses in participants with complete data (Web Table 5) and using the modified dietary diabetes risk-reduction score (Web Table 6).

**Table 2:** Hazard Ratios for Associations of Dietary Diabetes Risk-Reduction Score With All-Cause and Cause-Specific Mortality, a Post Hoc Analysis of the Prostate, Lung, Colorectal, and Ovarian Cancer Screening Trial, United States, 1993–2015

**Mortality Cause and the Ranges (Medians) of Quintiles of Dietary Diabetes Risk-Reduction Score**	**No. of Deaths**	**Death Rate** ^**a**^	**Model 1** ^**b**^		**Model 2** ^**c**^		**Model 3** ^**d**^		**Model 4** ^**e**^	
			**HR**	**95% CI**	**HR**	**95% CI**	**HR**	**95% CI**	**HR**	**95% CI**
All-cause mortality										
9–19 (17)	3,774	174.13	1.00	Referent	1.00	Referent	1.00	Referent	1.00	Referent
20–22 (21)	3,870	163.31	0.89	0.85, 0.93	0.93	0.89, 0.98	0.92	0.88, 0.97	0.90	0.86, 0.93
23–24 (23)	2,721	150.81	0.82	0.78, 0.86	0.88	0.84, 0.93	0.87	0.82, 0.91	0.83	0.79, 0.87
25–27 (26)	3,451	140.11	0.75	0.71, 0.78	0.83	0.79, 0.87	0.81	0.77, 0.85	0.82	0.78, 0.86
28–40 (30)	3,716	126.41	0.68	0.65, 0.71	0.79	0.76, 0.83	0.76	0.72, 0.80	0.74	0.70, 0.78
*P* for trend			<0.001		<0.001		<0.001		<0.001	
Cardiovascular mortality										
9–19 (17)	1,044	48.17	1.00	Referent	1.00	Referent	1.00	Referent	1.00	Referent
20–22 (21)	1,040	43.89	0.86	0.79, 0.93	0.90	0.83, 0.99	0.89	0.81, 0.97	0.85	0.78, 0.92
23–24 (23)	760	42.12	0.82	0.74, 0.90	0.89	0.81, 0.98	0.86	0.79, 0.95	0.80	0.73, 0.88
25–27 (26)	968	39.3	0.75	0.68, 0.82	0.84	0.77, 0.92	0.81	0.74, 0.89	0.82	0.75, 0.90
28–40 (30)	997	33.92	0.65	0.60, 0.71	0.79	0.72, 0.87	0.73	0.66, 0.81	0.69	0.62, 0.76
*P* for trend			<0.001		<0.001		<0.001		<0.001	
Cancer mortality										
9–19 (17)	1,219	56.24	1.00	Referent	1.00	Referent	1.00	Referent	1.00	Referent
20–22 (21)	1,285	54.23	0.95	0.87, 1.02	1.00	0.92, 1.08	0.99	0.92, 1.08	0.97	0.90, 1.04
23–24 (23)	873	48.39	0.85	0.78, 0.93	0.92	0.84, 1.00	0.91	0.83, 1.00	0.89	0.81, 0.97
25–27 (26)	1,120	45.47	0.80	0.73, 0.86	0.89	0.82, 0.97	0.88	0.81, 0.96	0.90	0.83, 0.98
28–40 (30)	1,222	41.57	0.74	0.68, 0.80	0.87	0.80, 0.94	0.85	0.78, 0.94	0.83	0.76, 0.92
*P* for trend			<0.001		<0.001		<0.001		<0.001	

Abbreviations: CI, confidence interval; HR, hazard ratio.

^a^ Crude death rate per 10,000 person-years.

^b^ Adjusted for age (years) and sex (male, female).

^c^ Adjustments from model 1 plus ethnicity (non-Hispanic White, non-Hispanic Black, Hispanic, others), trial arm (intervention, control), educational level (some college or below, college graduate, postgraduate), marital status (married or living as married, widowed, divorced, separated, never married), history of hypertension (yes, no), family history of cancer (yes, no; only for all-cause and cancer mortality), aspirin use (yes, no), single or multivitamin supplement use (yes, no), smoking status (current, past, never), alcohol consumption (g/day), body mass index, physical activity (minutes/week), and energy intake from diet (kcal/day).

^d^ Adjustments from model 2 plus consumption of fruits (g/day), vegetables (g/day), tea (g/day), fish (g/day), and dairy (servings/day).

^e^ Adjustments from model 2 plus consumption of fruits (g/day), vegetables (g/day), tea (g/day), fish (g/day), and dairy (servings/day), with body mass index treated as a time-varying covariate.

### Subgroup analyses

Interestingly, subgroup analyses found that the inverse association with cardiovascular mortality was more pronounced in women than in men (*P* for interaction = 0.024), whereas the inverse association with cancer mortality was more pronounced among men than women (*P* for interaction = 0.032) (Table 3 and Web Table 7). Moreover, the inverse associations with all-cause (*P* for interaction = 0.023) and cancer (*P* for interaction = 0.023) mortality were more pronounced among participants with heavy alcohol consumption than those with no, light, or moderate alcohol consumption. In addition, the inverse association with cancer mortality was more pronounced among current or past smokers than never smokers (*P* for interaction = 0.002). No significant interaction effect was found for the remaining stratification factors (all *P* for interaction > 0.05).

**Table 3:** Subgroup Analyses of the Association of Dietary Diabetes Risk-Reduction Score With All-Cause and Cause-Specific Mortality, a Post Hoc Analysis of the Prostate, Lung, Colorectal, and Ovarian Cancer Screening Trial, United States, 1993–2015

**Subgroup Variable**	**All-Cause Mortality**			**Cardiovascular Mortality**			**Cancer Mortality**		
	**HR** ^**a**^	**95% CI**	***P* for Interaction**	**HR** ^**a**^	**95% CI**	***P* for Interaction**	**HR** ^**a**^	**95% CI**	***P* for Interaction**
Age, years			0.345			0.781			0.825
≥65	0.77	0.72, 0.81		0.71	0.64, 0.80		0.86	0.77, 0.96	
<65	0.75	0.68, 0.83		0.81	0.65, 1.00		0.85	0.73, 0.99	
Sex			0.280			0.024			0.032
Male	0.77	0.72, 0.83		0.81	0.71, 0.92		0.78	0.69, 0.89	
Female	0.74	0.68, 0.80		0.61	0.52, 0.72		0.94	0.81, 1.08	
Trial group			0.592			0.199			0.469
Intervention	0.76	0.71, 0.82		0.78	0.68, 0.90		0.84	0.74, 0.95	
Control	0.76	0.71, 0.82		0.68	0.59, 0.79		0.87	0.77, 0.99	
History of hypertension			0.997			0.595			0.504
Yes	0.76	0.69, 0.82		0.73	0.62, 0.85		0.82	0.70, 0.97	
No	0.77	0.72, 0.82		0.74	0.65, 0.84		0.87	0.78, 0.97	
Body mass index^b^			0.104			0.114			0.299
≥25	0.79	0.74, 0.84		0.78	0.69, 0.88		0.88	0.79, 0.99	
<25	0.72	0.66, 0.78		0.66	0.55, 0.78		0.81	0.70, 0.95	
Smoking status			0.149			0.663			0.002
Current or past	0.70	0.66, 0.75		0.72	0.63, 0.81		0.73	0.65, 0.82	
Never	0.77	0.71, 0.84		0.69	0.59, 0.80		1.00	0.86, 1.17	
Alcohol intake, g/day^c^			0.023			0.895			0.023
Heavy	0.67	0.59, 0.77		0.83	0.63, 1.10		0.65	0.51, 0.82	
None, light, or moderate	0.79	0.75, 0.84		0.74	0.66, 0.82		0.90	0.81, 0.99	

Abbreviations: CI, confidence interval; HR, hazard ratio.

^a^ HRs are for the comparison of quintile 5 to quintile 1 and are adjusted for age (years), sex (male, female), ethnicity (non-Hispanic White, non-Hispanic Black, Hispanic, others), trial arm (intervention, control), educational level (some college or below, college graduate, postgraduate), marital status (married or living as married, widowed, divorced, separated, never married), history of hypertension (yes, no), family history of cancer (yes, no; only for all-cause and cancer mortality), aspirin use (yes, no), single or multivitamin supplement use (yes, no), smoking status (current, past, never), alcohol consumption (g/day), body mass index, physical activity (min/week), energy intake from diet (kcal/day), and consumption of fruits (g/day), vegetables (g/day), tea (g/day), fish (g/day), and dairy (servings/day). In subgroup analyses stratified by sex, trial arm, history of hypertension, and smoking status, HRs were not adjusted for the stratification factor.

^b^ Weight (kg)/height (m)^2^.

^c^ Light, moderate, and heavy alcohol consumption are defined as ≤6 g/day, >6 and <28 g/day for men and >6 and <14 g/day for women, and >28 g/day for men and >14 g/day for women, respectively.

### Sensitivity analyses

The initial associations of dietary diabetes risk-reduction score with risks of death from all causes, cardiovascular disease, and cancer did not change materially in a large range of sensitivity analyses (Web Table 8).

### **Associations by each component of type 2 diabetes**–**prevention diet**

Comparing quintile 5 to quintile 1, higher intake of cereal fiber (HR = 0.79, 95% CI: 0.74, 0.85; *P* for trend < 0.001), nuts (HR = 0.82, 95% CI: 0.78, 0.86; *P* for trend < 0.001), or coffee (HR = 0.88, 95% CI: 0.84, 0.93; *P* for trend< 0.001) was found to be associated with a lower risk of all-cause mortality, whereas higher intake of sugar-sweetened beverages (HR = 1.00, 95% CI: 0.95, 1.05; *P* for trend = 0.022) was found to be associated with a higher risk of all-cause mortality (Table 4); moreover, an inverse association was found for the ratio of polyunsaturated to saturated fatty acids and all-cause mortality (HR = 0.84, 95% CI: 0.80, 0.89; *P* for trend< 0.001). A marginally significant positive association was found for red and processed meat consumption and all-cause mortality (HR = 1.01, 95% CI: 0.95, 1.07; *P* for trend = 0.052). Similar results were obtained for cardiovascular and/or cancer mortality. No significant associations with mortality from all causes, cardiovascular disease, and cancer were found for glycemic index and *trans*-fatty acid intake.

**Table 4:** Association of Each Component of a Type 2 Diabetes–Prevention Diet With All-Cause and Cause-Specific Mortality, a Post Hoc Analysis of the Prostate, Lung, Colorectal, and Ovarian Cancer Screening Trial, United States, 1993–2015

**Dietary Component and Quintile Range**	**All-Cause Mortality**			**Cardiovascular Mortality**			**Cancer Mortality**		
	**No. of Deaths**	**HR** ^**a**^	**95% CI**	**No. of Deaths**	**HR** ^**a**^	**95% CI**	**No. of Deaths**	**HR** ^**a**^	**95% CI**
Glycemic index									
<50.09	3,453	1.00	Referent	946	1.00	Referent	1,111	1.00	Referent
50.09–52.56	3,352	0.93	0.88, 0.97	921	0.91	0.83, 1.00	1,069	0.95	0.87, 1.03
52.57–54.58	3,507	0.93	0.89, 0.98	947	0.90	0.81, 0.99	1,123	0.96	0.88, 1.05
54.59–56.93	3,418	0.89	0.84, 0.94	938	0.87	0.79, 0.96	1,160	0.97	0.88, 1.06
≥56.94	3,802	0.95	0.90, 1.01	1,057	0.94	0.85, 1.05	1,256	1.00	0.91, 1.10
*P* for trend		0.061			0.225			0.817	
Cereal fiber, g/day									
<6.60	3,944	1.00	Referent	1,074	1.00	Referent	1,283	1.00	Referent
6.60–9.27	3,559	0.90	0.86, 0.95	967	0.91	0.83, 0.99	1,177	0.94	0.86, 1.02
9.28–12.08	3,325	0.85	0.81, 0.90	944	0.90	0.81, 0.99	1,055	0.84	0.77, 0.92
12.09–16.16	3,330	0.82	0.78, 0.87	882	0.80	0.72, 0.89	1,093	0.85	0.77, 0.94
≥16.17	3,374	0.79	0.74, 0.85	942	0.80	0.70, 0.92	1,111	0.82	0.73, 0.94
*P* for trend		<0.001			0.001			0.003	
Ratio of polyunsaturated to saturated fatty acids									
<0.52	4,268	1.00	Referent	1,196	1.00	Referent	1,357	1.00	Referent
0.52–0.65	3,634	0.91	0.87, 0.95	1,012	0.88	0.81, 0.96	1,171	0.93	0.86, 1.01
0.66–0.78	3,392	0.88	0.84, 0.92	945	0.84	0.77, 0.92	1,132	0.94	0.87, 1.02
0.79–0.98	3,188	0.85	0.81, 0.90	801	0.73	0.66, 0.80	1,058	0.91	0.84, 1.00
≥0.99	3,050	0.84	0.80, 0.89	855	0.77	0.70, 0.85	1,001	0.91	0.84, 1.00
*P* for trend		<0.001			<0.001			0.097	
*Trans-*fatty acids, g/day									
<1.57	3,508	1.00	Referent	1,017	1.00	Referent	1,044	1.00	Referent
1.57–2.66	3,265	0.92	0.88, 0.97	914	0.90	0.82, 0.99	1,037	0.96	0.88, 1.05
2.67–3.95	3,428	0.95	0.90, 1.00	940	0.91	0.83, 1.01	1,102	0.99	0.90, 1.08
3.96–5.95	3,514	0.95	0.90, 1.00	903	0.86	0.78, 0.96	1,240	1.06	0.96, 1.17
≥5.96	3,817	0.97	0.91, 1.04	1,035	0.94	0.83, 1.07	1,296	1.02	0.90, 1.15
*P* for trend		0.830			0.570			0.383	
Sugar-sweetened beverages, g/day									
<15.94	3,519	1.00	Referent	973	1.00	Referent	1,107	1.00	Referent
15.94–48.84	3,462	0.92	0.88, 0.96	935	0.88	0.81, 0.97	1,142	0.99	0.90, 1.09
48.85–130.34	3,479	0.93	0.89, 0.98	970	0.92	0.84, 1.01	1,156	1.00	0.92, 1.09
130.35–339.08	3,521	0.97	0.93, 1.02	962	0.95	0.86, 1.04	1,128	0.99	0.91, 1.08
≥339.09	3,551	1.00	0.95, 1.05	969	0.99	0.90, 1.09	1,186	1.01	0.92, 1.10
*P* for trend		0.022			0.191			0.835	
Nuts, g/day									
<0.08	4,231	1.00	Referent	1,180	1.00	Referent	1,292	1.00	Referent
0.08–1.09	3,651	0.93	0.89, 0.97	1,021	0.94	0.86, 1.02	1,201	1.01	0.93, 1.09
1.10–3.41	3,284	0.84	0.81, 0.88	913	0.85	0.78, 0.93	1,038	0.87	0.80, 0.94
3.42–7.78	3,229	0.84	0.80, 0.88	849	0.80	0.73, 0.87	1,097	0.93	0.86, 1.01
≥7.79	3,137	0.82	0.78, 0.86	846	0.80	0.73, 0.88	1,091	0.92	0.84, 1.00
*P* for trend		<0.001			<0.001			0.163	
Coffee, g/day									
<24.17	3,307	1.00	Referent	960	1.00	Referent	948	1.00	Referent
24.17–441.20	3,454	0.91	0.87, 0.96	1,027	0.92	0.84, 1.00	987	0.94	0.86, 1.02
441.21–1,050.32	3,440	0.86	0.82, 0.90	922	0.79	0.72, 0.87	1,155	1.04	0.95, 1.13
1,050.33–1,278.36	3,360	0.83	0.79, 0.87	861	0.75	0.68, 0.83	1,200	1.03	0.94, 1.12
≥1,278.37	3,971	0.88	0.84, 0.93	1,039	0.82	0.74, 0.90	1,429	1.07	0.98, 1.17
*P* for trend		<0.001			<0.001			0.010	
Red and processed meat, g/day									
<2.19	3,212	1.00	Referent	946	1.00	Referent	900	1.00	Referent
2.19–5.01	3,246	0.96	0.91, 1.01	897	0.90	0.82, 0.99	1,041	1.09	1.00, 1.19
5.02–9.38	3,294	0.92	0.87, 0.97	861	0.81	0.74, 0.89	1,094	1.07	0.98, 1.17
9.39–18.58	3,660	0.96	0.91, 1.01	1,011	0.89	0.81, 0.98	1,266	1.16	1.06, 1.28
≥18.59	4,120	1.01	0.95, 1.07	1,094	0.89	0.80, 0.99	1,418	1.19	1.08, 1.31
*P* for trend		0.052			0.590			0.002	

Abbreviations: CI, confidence interval; HR, hazard ratio.

^a^ Adjusted for age (years), sex (male, female), ethnicity (non-Hispanic White, non-Hispanic Black, Hispanic, others), trial arm (intervention, control), educational level (college below, college graduate, postgraduate), marital status (married or living as married, widowed, divorced, separated, never married), history of hypertension (yes, no), family history of cancer (yes, no; only for all-cause and cancer mortality), aspirin use (yes, no), single or multivitamin supplement use (yes, no), smoking status (current, past, never), alcohol consumption (g/day), body mass index, physical activity (min/week), energy intake from diet (kcal/day), and consumption of fruits (g/day), vegetables (g/day), tea (g/day), fish (g/day), and dairy (servings/day). All individual components were mutually adjusted, with each component treated as a continuous variable in regression models.

## DISCUSSION

In this large prospective multicenter study with a mean follow-up of up to 13.6 years, we found that greater adherence to a type 2 diabetes–prevention diet, as indicated by higher dietary diabetes risk-reduction score, was associated with lower risks of death from all causes, cardiovascular disease, and cancer. Subgroup analyses further found that sex, smoking status, and alcohol consumption were effect modifiers of the observed associations between dietary diabetes risk-reduction score and risks of death from all causes, cardiovascular disease, and/or cancer.

Many previous studies in nutritional epidemiology focus on the roles of individual foods or nutrients in health outcomes. However, considering the potential antagonistic or synergistic effects among dietary components and the fact that individuals always consume a variety of foods simultaneously in their daily life, the health effects of a given dietary pattern may be different from the sum of its individual components (31). Therefore, dietary pattern evaluation possibly could provide a better understanding for the roles of diets in health outcomes. In fact, the advantages of analyzing the dietary pattern in the field of public health are increasingly being recognized. For example, the 2015 Dietary Guideline Advisory Committee made its dietary recommendations based on dietary patterns rather than individual foods or nutrients (33). A growing number of studies have shown favorable associations of healthy dietary patterns with mortality risk (34). For example, Patel et al. (35) recently found that adherence to Dietary Approaches to Stop Hypertension, the Alternate Healthy Eating Index, or the Mediterranean diet was associated with a decreased risk of all-cause mortality. In this secondary analysis of the PLCO Cancer Screening Trial, we have assessed, to our knowledge for the first time, the role of the type 2 diabetes–prevention diet in the risk of mortality and we found that adherence to this dietary pattern was associated with reduced risks of all-cause and cause-specific mortality. Our findings are consistent with those from previous studies (34, 35) and extend the favorable associations between healthy dietary pattern and mortality to a type 2 diabetes–prevention diet. Thus, our findings deepen our understanding of the role of dietary exposures in relation to the risk of type 2 diabetes in determining mortality risk. Meanwhile, our findings suggest that increasing intakes of cereal fiber, polyunsaturated fatty acids, coffee, and nuts while decreasing intakes of carbohydrates, *trans*-fatty acids, red and processed meat, and sugar-sweetened beverages may be helpful for improving longevity, which is particularly significant in that dietary behavior can be modifiable and unhealthy diet is a leading cause of mortality in the US population (3). In addition, our findings highlight the importance of adhering to a healthy dietary pattern in improving health outcomes and provide some supporting evidence for the recommendation of adhering to a healthy eating pattern by the 2015–2020 US dietary guidelines (36).

In this study, we observed inverse associations with all-cause or cause-specific mortality for the ratio of polyunsaturated to saturated fatty acids and intakes of cereal fiber, nuts, and coffee, a positive association for sugar-sweetened beverages, and a null association for glycemic index, which are consistent with the results of previous studies (10–14, 16, 37). However, our study revealed a null association between red and processed meat consumption and cardiovascular mortality, which is inconsistent with a recent prospective cohort study showing a significant positive association (for tertile 3 vs. tertile 1, HR = 1.33, 95% CI: 1.19, 1.49) (38). The inconsistency may be due to the difference in study population; that previous study was conducted among UK adults aged 40–69 years (38). It is also possible that the positive association of red and processed meat consumption with cardiovascular mortality observed in the previous study (37) was due to incomplete adjustment for known confounders (15), such as physical activity. In addition, our study observed a null association of *trans*-fatty acid intake with all-cause mortality, which is consistent with the results from a prospective study in a British working population (per 1-standard-deviation increase, HR = 1.07, 95% CI: 0.98, 1.18) (39) but is inconsistent with those from several studies showing a positive association (11, 40, 41). The exact reasons for the above phenomenon are unclear and may be attributable to the differences in study population, methodology, and/or the extent of adjustment for potential confounders. Hence, more studies are needed to investigate the associations of intakes of red and processed meat and *trans*-fatty acid with all-cause and cause-specific mortality.

Interestingly, our study observed that the inverse association with cardiovascular mortality was more pronounced in women than in men, while the inverse association with cancer mortality was more pronounced in men than in women, indicating that sex is an outcome-specific effect modifier in our study setting. The exact reasons for this observation are unclear; it may be related to hormonal differences between the sexes. As almost all women in this study were postmenopausal, estrogen-level difference between men and women is not expected to be a major driver for this observation. Instead, testosterone-level difference between the sexes may be a key inducer. Indeed, observational studies have found that testosterone replacement therapy is associated with an increased risk of cardiovascular events (42) but a decreased risk of aggressive prostate cancer (43); thus, the relatively high testosterone level in men may attenuate the inverse association of the dietary diabetes risk-reduction score with cardiovascular mortality but strengthen the inverse association with cancer mortality. In addition, our subgroup analyses found that the inverse association of dietary diabetes risk-reduction score with cancer mortality was more pronounced in current or past smokers or participants with heavy alcohol consumption, suggesting that diabetes-prevention diet may have interactions with smoking and alcohol drinking in biological pathways. In fact, a prospective cohort study also showed that the inverse association of adherence to Dietary Approaches to Stop Hypertension diet with the risk of all-cause mortality was more pronounced in smokers than in nonsmokers (44). Of note, we cannot rule out a possibility that the above-mentioned interactions are chance findings, although they are biologically possible. Therefore, our findings from subgroup analyses warrant further investigation.

Although the specific mechanisms underlying the inverse associations of the type 2 diabetes–prevention diet with risks of all-cause and cause-specific mortality remain to be explored, intuitively, this dietary pattern possibly exerts its mortality benefits through its individual components. Human and experimental studies have suggested that polyunsaturated fatty acids are capable of improving insulin resistance (45, 46). Moreover, coffee has been found to inhibit inflammatory responses, possibly by reducing the expression of inflammation-related genes (47) and the release of inflammatory mediators (48). In addition, nut consumption has been found to be associated with attenuated oxidative stress (49), which may be through the modulation of nuclear factor-kB and nuclear factor erythroid 2–related factor 2/heme oxygenase-1 pathways (50). Collectively, these facts suggest that the inverse association of the type 2 diabetes–prevention diet with mortality may be explained by improved insulin resistance and decreased levels of inflammation and oxidative stress. Nevertheless, it is also possible that mortality benefits of adhering to this diet are mediated, at least partly, by potential interactions among individual components of the diet.

Our study has several limitations. First, food consumption information used for the construction of dietary diabetes risk-reduction score was evaluated once at baseline in our study. As dietary habits can change over time, food consumption evaluation at 1 time point may result in nondifferential bias. Nonetheless, it has been suggested that the approaches using baseline diet data only in general yield a weaker association than do these using the cumulative averages (51). In addition, in this study, nutrient intake was assessed with the DHQ, a self-administered food frequency questionnaire. However, this questionnaire did not contain the essential information that was required to accurately calculate intakes of some nutrients. For example, some *trans* fats are artificial and added into processed food products. Thus, the content of *trans* fats in a food product may depend on the brand of the product. However, the DHQ did not contain this information. Hence, nutrient assessment by the DHQ might be subject to measurement errors. Second, death certificates were employed to obtain the underlying cause of mortality in our study. Of note, the cause of mortality from death certificates may be misclassified in some circumstances (52). Hence, our findings on the association of dietary diabetes risk-reduction score with cause-specific mortality might be susceptible to misclassification bias. Moreover, the validity of mortality assessment in the PLCO Cancer Screening Trial has not been confirmed, raising some concerns on the accuracy of outcome ascertainment. Third, in our study, all participants were US adults between the ages of 55 and 74 years; moreover, 90.9% of participants were non-Hispanic White, 36.6% were college graduates, and 51.0% were current or past smokers. Therefore, our findings may not be generalizable to other populations. Fourth, as shown in Table 4, not all dietary components were associated with all-cause or cause-specific mortality. However, when constructing diabetes risk-reduction score, we assumed that each component contributes equally to the score. Thus, the score used in our study may not precisely reflect the actual role of each dietary component in the real world. Finally, as with any observational study, our results might be influenced by residual confounding due to unmeasured or unrecognized confounders, although a wide range of potential confounders was controlled for. In addition, it should be acknowledged that, based on our findings, the causal association of adhering to a diabetes-prevention diet with mortality risk cannot be established, given the observational design of our study.

In conclusion, the dietary diabetes risk-reduction score is inversely associated with the risks of death from all causes, cardiovascular disease, and cancer in this US population. These findings suggest that adherence to a type 2 diabetes–prevention diet may serve as an attractive strategy for improving longevity. Future studies should clarify the relevant biological mechanisms and validate our findings in other populations.

## Supplementary Material

Web_Material_kwab265
