# Supplementary material for: Type 2 Diabetes–Prevention Diet and All-Cause and Cause-Specific Mortality: A Prospective Study
Source: Am J Epidemiol. 2021 Nov 2;191(3):472–86. doi: 10.1093/aje/kwab265 (PMC8895391; doi:10.1093/aje/kwab265)
Supplement: Web_Material_kwab265 [file web_material_kwab265.pdf]

**Web Material**

# Type 2 diabetes prevention diet and all-cause and cause-specific mortality in the US population: a prospective study

Chun-Rui Wang, Tian-Yang Hu, Fa-Bao Hao, Nan Chen, Yang Peng, Jing-Jing Wu, Peng-Fei Yang, and Guo-Chao Zhong

**Contents**

**Web Table 1.** Criteria for determining dietary diabetes risk reduction score

**Web Table 2.** Criteria for determining the modified dietary diabetes risk reduction score

**Web Table 3.** Distribution of covariates with missing data before and after imputation

**Web Table 4.** Absolute risk differences of all-cause and cause-specific mortality associated with dietary diabetes risk reduction score

**Web Table 5.** Hazard ratios of the association of dietary diabetes risk reduction score with all-cause and cause-specific mortality in 64322 participants with complete data

**Web Table 6.** Hazard ratios of association of the modified dietary diabetes risk reduction score with all-cause and cause-specific mortality

**Web Table 7.** ARDs for the association of dietary diabetes risk reduction score with all-cause and cause-specific mortality by stratification factors

**Web Table 8.** Sensitivity analyses on the association of dietary diabetes risk reduction score with all-cause and cause-specific mortality, the Prostate, Lung, Colorectal, and Ovarian Cancer Screening Trial

**Web Table 1.** Criteria for determining dietary diabetes risk reduction score, a post hoc analysis of the Prostate, Lung, Colorectal, and Ovarian Cancer Screening Trial, 1993-2015

| | Energy-adjusted dietary intakes of individual components | | | | | | | |
|---|---|---|---|---|---|---|---|---|
| Points | Cereal fiber (g/day) | Ratio of polyunsaturated to saturated fatty acids | Coffee (g/day) | Nuts (g/day) | Glycemic index | *Trans* fatty acids (g/day) | Red and processed meat (g/day) | Sugar-sweetened beverages (g/day) |
| 5 | ≥16.19 | ≥1.00 | ≥1278.38 | ≥7.80 | ≤50.08 | ≤1.57 | ≤2.19 | ≤15.94 |
| 4 | 12.10–16.18 | 0.80–0.99 | 1050.34–1278.37 | 3.43–7.79 | 50.09–52.57 | 1.58–2.67 | 2.20–5.02 | 15.95–48.85 |
| 3 | 9.29–12.09 | 0.67–0.79 | 441.22–1050.33 | 1.11–3.42 | 52.58–54.59 | 2.68–3.95 | 5.03–9.39 | 48.86–130.35 |
| 2 | 6.61–9.28 | 0.52–0.66 | 24.18–441.21 | 0.09–1.10 | 54.60–56.94 | 3.96–5.96 | 9.40–18.58 | 130.36–339.09 |
| 1 | ≤6.60 | ≤0.51 | ≤24.17 | ≤0.08 | ≥56.95 | ≥5.97 | ≥18.59 | ≥339.10 |

**Web Table 2.** Criteria for determining the modified dietary diabetes risk reduction score, a post hoc analysis of the Prostate, Lung, Colorectal, and Ovarian Cancer Screening Trial, 1993-2015

| | Energy-adjusted dietary intakes of individual components | | | | | | | | | |
|---|---|---|---|---|---|---|---|---|---|---|
| Points | Fruits (g/day) | Vegetables (g/day) | Cereal fiber (g/day) | Ratio of polyunsaturated to saturated fatty acids | Coffee (g/day) | Nuts (g/day) | Glycemic index | *Trans* fatty acids (g/day) | Red and processed meat (g/day) | Sugar-sweetened beverages (g/day) |
| 5 | ≥396.60 | ≥392.95 | ≥16.19 | ≥1.00 | ≥1278.38 | ≥7.80 | ≤50.08 | ≤1.57 | ≤2.19 | ≤15.94 |
| 4 | 275.71, 396.59 | 280.19, 392.94 | 12.10, 16.18 | 0.80, 0.99 | 1050.34, 1278.37 | 3.43, 7.79 | 50.09, 52.57 | 1.58, 2.67 | 2.20, 5.02 | 15.95, 48.85 |
| 3 | 187.23, 275.70 | 207.10, 280.18 | 9.29, 12.09 | 0.67, 0.79 | 441.22, 1050.33 | 1.11, 3.42 | 52.58, 54.59 | 2.68, 3.95 | 5.03, 9.39 | 48.86, 130.35 |
| 2 | 106.58, 187.22 | 141.35, 207.09 | 6.61, 9.28 | 0.52, 0.66 | 24.18, 441.21 | 0.09, 1.10 | 54.60, 56.94 | 3.96, 5.96 | 9.40, 18.58 | 130.36, 339.09 |
| 1 | ≤106.57 | ≤141.34 | ≤6.60 | ≤0.51 | ≤24.17 | ≤0.08 | ≥56.95 | ≥5.97 | ≥18.59 | ≥339.10 |

**Web Table 3.** Distribution of covariates with missing data before and after imputation, a post hoc analysis of the Prostate, Lung, Colorectal, and Ovarian Cancer Screening Trial, 1993-2015 [a]

| Covariate | Before imputation | After imputation | Number (%) with missing data |
|---|---|---|---|
| Aspirin use | | | |
| Yes | 37642 (43.6) | 37642 (43.4) | 369 (0.43) |
| No | 48622 (56.4) | 48991 (56.6) | |
| Educational level | | | |
| College below | 54374 (62.9) | 54479 (62.9) | 175 (0.20) |
| College graduate | 15516 (17.9) | 15567 (18.0) | |
| Postgraduate | 16568 (19.2) | 16587 (19.1) | |
| Marital status | | | |
| Married or living as married | 67832 (78.4) | 68133 (78.6) | 165 (0.19) |
| Widowed | 6893 (8.0) | 6858 (7.9) | |
| Divorced | 8366 (9.7) | 8296 (9.6) | |
| Separated | 654 (0.8) | 649 (0.7) | |
| Never married | 2723 (3.1) | 2697 (3.1) | |
| History of hypertension | | | |
| Yes | 24852 (28.9) | 25082 (29.0) | 506 (0.58) |
| No | 61275 (71.1) | 61551 (71.0) | |
| Smoking status | | | |
| Current | 8059 (9.3) | 7999 (9.2) | 18 (0.02) |
| Past | 35893 (41.4) | 35933 (41.5) | |
| Never | 42663 (49.3) | 42701 (49.3) | |
| Family history of cancer | | | |
| Yes | 48611 (56.3) | 48851 (56.4) | 240 (0.28) |
| No | 37782 (43.7) | 37782 (43.6) | |
| Body mass index (kg/m$^2$) | 27.0 ± 4.7 | 27.0 ± 4.7 | 1137 (1.31) |
| Physical activity level (min/week) [b] | 126.7 ± 122.6 | 124.2 ± 123.0 | 20723 (23.92) |

[a] Values are mean (standard deviation) or counts (percentage) as indicated.

[b] Total time of moderate-to-vigorous physical activity per week.

**Web Table 4.** Absolute risk differences of all-cause and cause-specific mortality associated with dietary diabetes risk reduction score, a post hoc analysis of the Prostate, Lung, Colorectal, and Ovarian Cancer Screening Trial, 1993-2015 [a]

| Causes of mortality | Quintiles of dietary diabetes risk reduction score, range (median) | | | | |
|---|---|---|---|---|---|
| | 9–19 (17) | 20–22 (21) | 23–24 (23) | 25–27 (26) | 28–40 (30) |
| All-causes | 0.00 (reference) | -15.14 (-23.75, -5.39) | -26.02 (-38.22, -17.22) | -40.85 (-52.01, -30.73) | -81.94 (-93.76, -71.12) |
| Cardiovascular disease | 0.00 (reference) | -5.95 (-11.30, -1.49) | -7.84 (-12.80, -2.54) | -11.30 (-16.92, -5.95) | -17.82 (-24.81, -11.30) |
| Cancer | 0.00 (reference) | -0.57 (-4.89, 4.17) | -5.56 (-11.52, 0.00) | -7.67 (-13.19, -2.34) | -9.92 (-15.86, -3.59) |

[a] Values were absolute risk differences (95% confidence intervals) in mortality rates per 10000 person-years, and adjusted for age (years), sex (male, female), ethnicity (non-Hispanic white, non-Hispanic black, Hispanic, others), trial arm (intervention, control), educational level (college below, college graduate, postgraduate), marital status (married or living as married, widowed, divorced, separated, never married), history of hypertension (yes, no), family history of cancer (yes, no; only for all-cause and cancer mortality), aspirin use (yes, no), single or multivitamin supplement use (yes, no), smoking status (current, past, never), alcohol consumption (g/day), body mass index (kg/m$^2$), physical activity (min/week), energy intake from diet (kcal/day), and consumption of fruits (g/day), vegetables (g/day), tea (g/day), fish (g/day), and dairy (servings/day).

**Web Table 5.** Hazard ratios of the association of dietary diabetes risk reduction score with all-cause and cause-specific mortality in 64322 participants with complete data, a post hoc analysis of the Prostate, Lung, Colorectal, and Ovarian Cancer Screening Trial, 1993-2015 [a]

| Causes of mortality | Quintiles of dietary diabetes risk reduction score, range (median) | | | | | $P_{trend}$ |
|---|---|---|---|---|---|---|
| | 9–19 (18) | 20–22 (21) | 23–24 (23) | 25–27 (26) | 28–40 (30) | |
| All causes | | | | | | |
| Deaths | 1923 | 2068 | 1466 | 1887 | 2050 | |
| Death rate [b] | 116.79 | 112.95 | 104.64 | 97.96 | 88.62 | |
| Model 1 [c] | 1.00 (reference) | 0.92 (0.87, 0.98) | 0.85 (0.80, 0.91) | 0.78 (0.73, 0.83) | 0.71 (0.67, 0.76) | <0.001 |
| Model 2 [d] | 1.00 (reference) | 0.97 (0.91, 1.03) | 0.92 (0.85, 0.98) | 0.87 (0.81, 0.92) | 0.84 (0.78, 0.90) | <0.001 |
| Model 3 [e] | 1.00 (reference) | 0.96 (0.90, 1.02) | 0.90 (0.84, 0.97) | 0.85 (0.79, 0.91) | 0.81 (0.76, 0.87) | <0.001 |
| Cardiovascular disease | | | | | | |
| Deaths | 522 | 553 | 400 | 501 | 557 | |
| Death rate [b] | 31.70 | 30.20 | 28.55 | 26.01 | 24.08 | |
| Model 1 [c] | 1.00 (reference) | 0.90 (0.80, 1.02) | 0.85 (0.74, 0.97) | 0.75 (0.66, 0.85) | 0.70 (0.62, 0.79 | <0.001 |
| Model 2 [d] | 1.00 (reference) | 0.94 (0.84, 1.07) | 0.91 (0.80, 1.04) | 0.84 (0.74, 0.95) | 0.84 (0.74, 0.95) | 0.0014 |
| Model 3 [e] | 1.00 (reference) | 0.92 (0.81, 1.04) | 0.88 (0.77, 1.00) | 0.79 (0.69, 0.90) | 0.76 (0.66, 0.87) | <0.001 |
| Cancer | | | | | | |
| Deaths | 615 | 691 | 480 | 618 | 694 | |
| Death rate [b] | 37.35 | 37.74 | 34.26 | 32.08 | 30.00 | |
| Model 1 [c] | 1.00 (reference) | 0.99 (0.89, 1.11) | 0.90 (0.80, 1.02) | 0.84 (0.75, 0.94) | 0.79 (0.71, 0.88) | <0.001 |
| Model 2 [d] | 1.00 (reference) | 1.04 (0.93, 1.16) | 0.96 (0.85, 1.09) | 0.92 (0.82, 1.04) | 0.91 (0.81, 1.03) | 0.033 |
| Model 3 [e] | 1.00 (reference) | 1.04 (0.93, 1.16) | 0.96 (0.85, 1.09) | 0.92 (0.82, 1.04) | 0.91 (0.80, 1.03) | 0.046 |

[a] Values are hazard ratios (95% confidence intervals).

[b] Crude death rate per 10000 person-years.

[c] Adjusted for age (years) and sex (male, female).

[d] Adjusted for model 1 plus ethnicity (non-Hispanic white, non-Hispanic black, Hispanic, others), trial arm (intervention, control), educational level (college below, college graduate, postgraduate), marital status (married or living as married, widowed, divorced, separated, never married), history of hypertension (yes, no), family history of cancer (yes, no; only for all-cause and cancer mortality), aspirin use (yes, no), single or multivitamin supplement use (yes, no), smoking status (current, past, never), alcohol consumption (g/day), body mass index (kg/m$^2$), physical activity (min/week), and energy intake from diet (kcal/day).

[e] Adjusted for model 2 plus consumption of fruits (g/day), vegetables (g/day), and tea (g/day), fish (g/day), and dairy (servings/day).

**Web Table 6.** Hazard ratios of association of the modified dietary diabetes risk reduction score with all-cause and cause-specific mortality, a post hoc analysis of the Prostate, Lung, Colorectal, and Ovarian Cancer Screening Trial, 1993-2015 [a]

| Causes of mortality | Quintiles of the modified dietary diabetes risk reduction score, range (median) | | | | | $P_{trend}$ |
|---|---|---|---|---|---|---|
| | 11–25 (23) | 26–28 (27) | 29–31 (30) | 32–35 (33) | 36–50 (38) | |
| **All causes** | | | | | | |
| Deaths | 4712 | 3296 | 3268 | 3322 | 2934 | |
| Death rate [b] | 171.54 | 159.33 | 148.49 | 138.04 | 126.41 | |
| Model 1 [c] | 1.00 (reference) | 0.88 (0.84, 0.92) | 0.80 (0.77, 0.84) | 0.73 (0.70, 0.76) | 0.68 (0.65, 0.71) | <0.001 |
| Model 2 [d] | 1.00 (reference) | 0.94 (0.90, 0.98) | 0.89 (0.85, 0.93) | 0.83 (0.79, 0.87) | 0.83 (0.79, 0.87) | <0.001 |
| **Cardiovascular disease** | | | | | | |
| Deaths | 1292 | 885 | 901 | 939 | 792 | |
| Death rate [b] | 47.03 | 42.78 | 40.94 | 39.02 | 34.12 | |
| Model 1 [c] | 1.00 (reference) | 0.86 (0.79, 0.93) | 0.80 (0.73, 0.87) | 0.74 (0.68, 0.81) | 0.66 (0.60, 0.72) | <0.001 |
| Model 2 [d] | 1.00 (reference) | 0.92 (0.84, 1.00) | 0.90 (0.82, 0.98) | 0.86 (0.79, 0.94) | 0.84 (0.76, 0.92) | <0.001 |
| **Cancer** | | | | | | |
| Deaths | 1518 | 1106 | 1051 | 1094 | 950 | |
| Death rate [b] | 55.26 | 53.46 | 47.76 | 45.46 | 40.93 | |
| Model 1 [c] | 1.00 (reference) | 0.95 (0.88, 1.02) | 0.84 (0.78, 0.91) | 0.79 (0.73, 0.86) | 0.73 (0.67, 0.79) | <0.001 |
| Model 2 [d] | 1.00 (reference) | 1.01 (0.94, 1.09) | 0.93 (0.85, 1.00) | 0.91 (0.84, 0.98) | 0.88 (0.81, 0.96) | 0.001 |

[a] Values are hazard ratios (95% confidence intervals).

[b] Crude death rate per 10000 person-years.

[c] Adjusted for age (years) and sex (male, female).

[d] Adjusted for model 1 plus ethnicity (non-Hispanic white, non-Hispanic black, Hispanic, others), trial arm (intervention, control), educational level (college below, college graduate, postgraduate), marital status (married or living as married, widowed, divorced, separated, never married), history of hypertension (yes, no), family history of cancer (yes, no; only for all-cause and cancer mortality), aspirin use (yes, no), single or

multivitamin supplement use (yes, no), smoking status (current, past, never), alcohol consumption (g/day), body mass index (kg/$m^2$), physical activity (min/week), and energy intake from diet (kcal/day).

**Web Table 7.** ARDs for the association of dietary diabetes risk reduction score with all-cause and cause-specific mortality by stratification factors, a post hoc analysis of the Prostate, Lung, Colorectal, and Ovarian Cancer Screening Trial, 1993-2015 [a]

| Subgroup variable | All-cause mortality | Cardiovascular mortality | Cancer mortality |
|---|---|---|---|
| | ARD $_{\text{quintile 5 } \textit{versus} \text{ 1}}$ (95% CI) | ARD $_{\text{quintile 5 } \textit{versus} \text{ 1}}$ (95% CI) | ARD $_{\text{quintile 5 } \textit{versus} \text{ 1}}$ (95% CI) |
| Age (years) | | | |
| ≥65 | -80.31 (-104.56, -63.07) | -31.59 (-43.50, -19.33) | -12.45 (-22.85, -3.19) |
| <65 | -33.20 (-46.87, -20.40) | -5.92 (-13.59, 0.00) | -7.12 (-14.91, -0.41) |
| Sex | | | |
| Male | -61.58 (-80.17, -42.22) | -13.68 (-23.82, -5.07) | -18.54 (-29.54, -8.13) |
| Female | -44.98 (-60.24, -32.01) | -21.45 (-30.97, -13.05) | -2.72 (-9.98, 3.15) |
| Trial group | | | |
| Intervention | -57.08 (-73.82, -39.67) | -13.67 (-22.81, -5.39) | -11.19 (-20.64, -3.09) |
| Control | -55.11 (-71.29, -38.31) | -23.44 (-34.61, -13.24) | -8.37 (-16.73, -0.57) |
| History of hypertension | | | |
| Yes | -66.59 (-90.74, -46.29) | -24.99 (-41.41, -11.92) | -12.71 (-24.81, -1.79) |
| No | -47.08 (-61.30, -34.60) | -13.86 (-21.24, -7.51) | -8.29 (-15.65, -1.72) |
| Body mass index (kg/m$^2$) | | | |
| ≥25 | -44.38 (-58.67, -31.80) | -13.72 (-21.85, -6.63) | -7.44 (-14.51, -0.55) |
| <25 | -75.20 (-99.61, -54.54) | -24.18 (-38.41, -13.24) | -14.24 (-26.02, -3.20) |
| Smoking status | | | |
| Current or past | -94.47 (-113.55, -73.48) | -22.88 (-34.56, -13.80) | -28.49 (-41.48, -16.91) |
| Never | -37.95 (-51.89, -24.20) | -16.77 (-25.93, -9.33) | 0.00 (-5.71, 5.10) |
| Alcohol consumption (g/day) [b] | | | |
| Heavy | -104.73 (-147.76, -63.51) | -10.02 (-28.72, 4.45) | -41.42 (-73.90, -16.88) |
| No, light, or moderate | -45.35 (-56.87, -32.50) | -16.90 (-24.78, -10.56) | -6.04 (-12.75, -0.55) |

Abbreviations: ARD, absolute risk difference; CI, confidence interval.

[a] Values were ARDs (95% confidence intervals) in mortality rates per 10000 person-years, and adjusted for age (years), sex (male, female), ethnicity (non-Hispanic white, non-Hispanic black, Hispanic, others), trial arm (intervention, control), educational level (college below, college graduate, postgraduate), marital status (married or living as married, widowed, divorced, separated, never married), history of hypertension (yes, no), family history of cancer (yes, no; only for all-cause and cancer mortality), aspirin use (yes, no), single or multivitamin supplement use (yes, no), smoking status (current, past, never), alcohol consumption (g/day), body mass index (kg/$m^2$), physical activity (min/week), energy intake from diet (kcal/day), and consumption of fruits (g/day), vegetables (g/day), tea (g/day), fish (g/day), and dairy (servings/day). In subgroup analyses stratified by sex, trial arm, history of hypertension, and smoking status, hazard ratios were not adjusted for the stratification factor.

[b] Light, moderate, and heavy alcohol consumption are defined as ≤6 g/day, >6–28 g/day for male and >6–14 g/day for female, and >28 g/day for male and >14 g/day for female, respectively.

**Web Table 8.** Sensitivity analyses on the association of dietary diabetes risk reduction score with all-cause and cause-specific mortality, a post hoc analysis of the Prostate, Lung, Colorectal, and Ovarian Cancer Screening Trial, 1993-2015 [a]

| Categories | Quintiles of dietary diabetes risk reduction score | | | | | $P_{trend}$ |
|---|---|---|---|---|---|---|
| | Quintile 1 | Quintile 2 | Quintile 3 | Quintile 4 | Quintile 5 | |
| All-cause mortality | | | | | | |
| Included participants with a history of stroke, heart attack, cancer, or diabetes [b] | 1.00 (reference) | 0.92 (0.89, 0.95) | 0.88 (0.85, 0.92) | 0.83 (0.80, 0.86) | 0.77 (0.74, 0.80) | <0.001 |
| Excluded deaths observed within the first five years of follow-up | 1.00 (reference) | 0.94 (0.89, 0.99) | 0.89 (0.84, 0.94) | 0.82 (0.78, 0.87) | 0.79 (0.74, 0.83) | <0.001 |
| Excluded participants with extreme values of energy intake [c] | 1.00 (reference) | 0.92 (0.88, 0.96) | 0.87 (0.83, 0.92) | 0.81 (0.77, 0.85) | 0.76 (0.72, 0.80) | <0.001 |
| Adjusted for propensity score on unadjusted model [d] | 1.00 (reference) | 0.94 (0.90, 0.98) | 0.87 (0.83, 0.92) | 0.81 (0.78, 0.85) | 0.75 (0.71, 0.78) | <0.001 |
| Further adjusted for Healthy Eating Index-2015 [e] | 1.00 (reference) | 0.98 (0.93, 1.03) | 0.95 (0.90, 1.00) | 0.92 (0.87, 0.97) | 0.91 (0.86, 0.97) | 0.001 |
| Further adjusted for plant-based diet index [e] | 1.00 (reference) | 0.93 (0.89, 0.98) | 0.88 (0.84, 0.93) | 0.83 (0.79, 0.87) | 0.78 (0.74, 0.83) | <0.001 |
| Further adjusted for intakes of polyunsaturated and saturated fatty acids [e] | 1.00 (reference) | 0.94 (0.90, 0.98) | 0.89 (0.84, 0.94) | 0.84 (0.79, 0.88) | 0.80 (0.76, 0.85) | <0.001 |
| Cardiovascular mortality | | | | | | |
| Included participants with a history of stroke, heart attack, cancer, or diabetes [b] | 1.00 (reference) | 0.91 (0.85, 0.97) | 0.87 (0.80, 0.93) | 0.83 (0.77, 0.89) | 0.73 (0.68, 0.79) | <0.001 |
| Excluded deaths observed within the first five years of follow-up | 1.00 (reference) | 0.92 (0.83, 1.01) | 0.91 (0.82, 1.01) | 0.82 (0.74, 0.91) | 0.76 (0.68, 0.84) | <0.001 |
| Excluded participants with extreme values of energy intake [c] | 1.00 (reference) | 0.89 (0.81, 0.97) | 0.87 (0.78, 0.95) | 0.81 (0.73, 0.89) | 0.73 (0.66, 0.81) | <0.001 |

| | | | | | | |
|---|---|---|---|---|---|---|
| Repeated analysis with competing risk regression model | 1.00 (reference) | 0.89 (0.82, 0.97) | 0.87 (0.80, 0.96) | 0.82 (0.75, 0.90) | 0.75 (0.68, 0.82) | <0.001 |
| Adjusted for propensity score on unadjusted model [d] | 1.00 (reference) | 0.91 (0.84, 0.99) | 0.88 (0.80, 0.97) | 0.83 (0.76, 0.90) | 0.73 (0.67, 0.79) | <0.001 |
| Further adjusted for Healthy Eating Index-2015 [e] | 1.00 (reference) | 0.95 (0.87, 1.04) | 0.96 (0.87, 1.06) | 0.93 (0.84, 1.03) | 0.90 (0.80, 1.01) | 0.069 |
| Further adjusted for plant-based diet index [e] | 1.00 (reference) | 0.90 (0.82, 0.98) | 0.88 (0.80, 0.97) | 0.83 (0.75, 0.91) | 0.76 (0.68, 0.84) | <0.001 |
| Further adjusted for intakes of polyunsaturated and saturated fatty acids [e] | 1.00 (reference) | 0.91 (0.84, 1.00) | 0.90 (0.82, 1.00) | 0.86 (0.78, 0.94) | 0.80 (0.72, 0.89) | <0.001 |
| Cancer mortality | | | | | | |
| Included participants with a history of stroke, heart attack, cancer, or diabetes [b] | 1.00 (reference) | 0.97 (0.91, 1.04) | 0.95 (0.88, 1.02) | 0.89 (0.83, 0.95) | 0.87 (0.81, 0.94) | <0.001 |
| Excluded deaths observed within the first five years of follow-up | 1.00 (reference) | 1.00 (0.91, 1.09) | 0.93 (0.84, 1.03) | 0.88 (0.80, 0.97) | 0.87 (0.79, 0.97) | 0.001 |
| Excluded participants with extreme values of energy intake [c] | 1.00 (reference) | 0.99 (0.91, 1.07) | 0.91 (0.83, 1.00) | 0.88 (0.81, 0.96) | 0.85 (0.78, 0.93) | <0.001 |
| Repeated analysis with competing risk regression model | 1.00 (reference) | 0.98 (0.91, 1.07) | 0.90 (0.82, 0.98) | 0.86 (0.80, 0.94) | 0.83 (0.76, 0.90) | <0.001 |
| Adjusted for propensity score on unadjusted model [d] | 1.00 (reference) | 0.96 (0.89, 1.04) | 0.87 (0.79, 0.94) | 0.82 (0.75, 0.88) | 0.76 (0.70, 0.82) | <0.001 |
| Further adjusted for Healthy Eating Index-2015 [e] | 1.00 (reference) | 1.03 (0.95, 1.12) | 0.95 (0.87, 1.05) | 0.92 (0.85, 1.00) | 0.90 (0.83, 0.99) | 0.033 |
| Further adjusted for plant-based diet index [e] | 1.00 (reference) | 1.00 (0.92, 1.08) | 0.92 (0.84, 1.01) | 0.89 (0.81, 0.97) | 0.86 (0.79, 0.95) | <0.001 |
| Further adjusted for intakes of | 1.00 (reference) | 1.01 (0.93, 1.09) | 0.93 (0.85, 1.02) | 0.91 (0.83, 0.99) | 0.89 (0.81, 0.98) | 0.005 |

polyunsaturated and saturated fatty acids [e]

[a] Values are hazard ratios (95% confidence intervals). Hazard ratios were adjusted for the following variables unless otherwise specified: age (years), sex (male, female), ethnicity (non-Hispanic white, non-Hispanic black, Hispanic, others), trial arm (intervention, control), educational level (college below, college graduate, postgraduate), marital status (married or living as married, widowed, divorced, separated, never married), history of hypertension (yes, no), family history of cancer (yes, no; only for all-cause and cancer mortality), aspirin use (yes, no), single or multivitamin supplement use (yes, no), smoking status (current, past, never), alcohol consumption (g/day), body mass index (kg/m$^2$), physical activity (min/week), energy intake from diet (kcal/day), and consumption of fruits (g/day), vegetables (g/day), tea (g/day), fish (g/day), and dairy (servings/day).

[b] Hazard ratios were additionally adjusted for history of stroke (yes, no), history of heart attack (yes, no), history of cancer (yes, no; only for all-cause and cancer mortality), and history of diabetes (yes, no).

[c] Extreme values of energy intake are defined as <800 or >4000 kcal/d for men and <500 or >3500 kcal/d for women.

[d] This covariate was treated as the continuous variable in multivariable Cox regression.

[‖] These covariates were treated as the continuous variable in multivariable Cox regression.
